# Anti-Phthisis Activities in Berkshire

**Published:** 1914-03-28

**Authors:** Arthur H. Norris

**Affiliations:** Tuberculosis Officer, Berkshire County Council. Shire Hall, Reading


### THE EDITOR'S LETTER-BOX.

### Anti-Phthisis Activities in Berkshire.

*To the Editor of* The Hospital.

Sir,—In a leading article in your issue of March 14 you comment on the recommendations of the Berkshire County Council for dealing with tuberculosis, stating "the county council sub-committees, as far as they have gone at present, do not favour the initiation immediately of a complete scheme, which must involve a considerable burden on the rates, but suggest the continuance and development of the present 'dispensary' scheme."

This is hardly a correct statement of the position. On the recommendation of the public health and education committees the county council has adopted a complete scheme for dealing with all classes of the community. This embraces the provision of sanatorium, hospital and observation beds, a sanatorium school for children, and a block grant for treatment of surgical tuberculosis. In addition to this there is the development of the dispensary system mentioned in your article. The scheme has received the approval of the Local Government Board, and will probably be in full working order before the autumn. The annual cost which will fall on the rates is estimated to be about £3,800 a year, which is equal to a three-farthing rate.

I shall be obliged if you will insert this, and thus remove a somewhat inaccurate impression which your article may have conveyed, for as a matter of fact the amount of sanatorium accommodation amounts to thirty beds at Peppard Sanatorium, sixteen hospital and observation beds at Abingdon, and thirty beds in a sanatorium school also at Peppard, seventeen of which are already in occupation, and are recognised as part of the joint scheme for Buckinghamshire and Berkshire. It is, however, quite true to say that the complete scheme only received the official sanction of the county council on the date of the issue in which the article appeared.—Yours faithfully,

Arthur H. Norris, M.R.C.S., D.P.H.,
Tuberculosis Officer, Berkshire County Council.

Shire Hall, Reading,
March 25, 1914.

